# A ^99m^Tc-tricine-HYNIC-labeled Peptide Targeting the Melanocortin-1 Receptor for Melanoma Imaging

**Published:** 2016

**Authors:** Danial Shamshirian, Mostafa Erfani, Davood Beiki, Maliheh Hajiramazanali, Babak Fallahi

**Affiliations:** a *Department of Radiopharmacy, School of Pharmacy, Tehran University of Medical Sciences, Tehran, Iran. *; b *Radiation Application Research School, Nuclear Science and Technology Research Institute (NSTRI), Tehran, Iran.*; c *Research Center for Nuclear Medicine, Tehran University of Medical Sciences, Tehran, Iran.*

**Keywords:** α-MSH, ^99^mTc, Tricine, HYNIC, Radiopeptide

## Abstract

Melanocortin-1 (MC1) receptor is an attractive melanoma-specific target for the development of α-MSH peptide based imaging and therapeutic agents. In this work a new lactam bridge α-MSH analogue was synthesized and radiolabeled with ^99m^Tc via HYNIC chelator and tricine as co-ligand. Also, stability in human serum, receptor bound internalization and tissue biodistribution in tumor bearing nude mice were thoroughly investigated. Radiolabeling with ^99m^Tc was performed at high specific activities (163MBq/nmol) with an acceptable labeling yield (>98%). The radioligand showed specific internalization into B16/F10 cells (13.35 ± 0.9% at 4 h). In biodistribution studies, a receptor-specific uptake was observed in MC1 receptor positive organ so that after 4 h the tumor uptake was 4.51 ± 0.11 % ID/g. Predominant renal excretion pathway with a highest accumulation of activity in tumor was observed for this radiopeptide. Obtained results show that the new designed labeled peptide conjugate can be a suitable candidate for diagnosis of metastatic melanomas.

## Introduction

Tumor imaging through peptide receptor targeting began over twenty years ago when radiolabelled somatostatin analogs were introduced into nuclear medicine for imaging of human tumors using a gamma camera. Since then, the use of radiolabeled peptides in nuclear medicine has been an important topic with increasing interest (1, 2). Up to now, several types of peptides have been introduced for tumor targeting and many types of cancer cells were demonstrated to overexpress various peptide receptors (3, 4). 

Alpha-melanocyte stimulating hormone (α-MSH) is a tridecapeptide (Ac-Ser-Tyr-Ser-Met-Glu-His-Phe-Arg-Trp-Gly-Lys-Pro-Val-NH_2_) produced in the pituitary gland, that binds to melanocortin-1 receptor (MC1R) on the surface of normal melanocytes, for the purpose of regulating melanin production involved with skin pigmentation. MC1R, was shown to be over-expressed on the surface of melanoma cells that derive form melanocyte cells (5-7). Over 80% of human metastatic melanomas were reported to overexpress MC1 receptors. Since melanoma causemore than 75% of deaths from skin cancer (8-10), There is a need to develop new radiopharmaceuticals which allowdiagnosingthis type of tumor in an early phase.

The main drawback of native α-MSH, is its extremely short biological half-life as it is easily hydrolyzed by proteases, and in addition the methionine residue in α-MSH can be oxidized (11). The sequence His-Phe-Arg-Trp, is slightly more stable and contains the amino acids which are essential for binding to MC1 receptors (12). It is also metabolized in plasma by endogenous proteases, thus reducing the biological activity in a physiologic surrounding (11). Newer generation agents have been designed to enhance their potency toward MC1R, including peptides with linear structures (13-15) and cyclic structures (16).

Recently, several new ^99m^Tc-α-MSH tracers have been reported (17), in which the radiolabeling step was achieved using a conjugate group such as hydrazino nicotinic acid (HYNIC), an N_3_S core and tricarbonyl (17, 18). Some of the technical limitations associated with these labeling methods have included low radiochemical yield, extended reaction times, as well as higher temperatures or pH of the reaction mixture, and lipophilicity of the chelator ligand. An important bifunctional chelating ligand is HYNIC, first reported to prepare ^99m^Tc-HYNIC-IgG, and since then, it has been conjugated to various biomolecules such as antibodies and peptides (19). Since HYNIC can only provide one or two coordination positionsto the radionuclide, co-ligands are necessary to complete the coordination sphere of the technetium core (20).

Guo *et al*. (17), recently evaluated the radiochemical and biological behavior of ^99m^Tc-HYNIC-cyclic-α-MSH analogue, andthey concluded the HYNIC moiety in the radioactive molecule promoted its stability as well as enhancing renal excretion. A similar methodology was used to evaluate ^99m^Tc-HYNIC-somatostatin, -bombesin, -substance P and -ubiquicidin analogues (21-24). 

Our efforts to pursue a ^99m^Tc-labeled peptide for tumor targeting, has led us to formulate a tricine complex of ^99m^Tc-HYNIC-Nle-CycMSH_hept _(Asp-His-DPhe-Arg-Trp-Gly-Lys) peptide containing gamma aminobutyric acid (GABA) as a three-carbon chain spacer between HYNIC and the N-terminus of the cyclic peptide. This study presents the synthesis of [HYNIC-GABA-Nle]-CycMSH_hept_, the optimum radiolabeling conditions with ^99m^Tc, and further characterization of the radiotracer using an *in-vivo* mouse tumor model. 

## Experimental

*General*

Sieber amide resin and all Fmoc-protected amino acids were commercially available (NovaBiochem; Switzerland).The prochelator HYNIC-Boc was synthesized according to Abrams et al (19). Other reagents were purchased (Merck; Germany) and used without further purification. All aqueous solutions were made using double distilled ultrapure water (<0.07 μS/cm) and filtered by 0.22 μ filters before biological uses. The cell-culture medium was Roswell Park Memorial Institute (RPMI-1640) supplemented with 10% fetal bovine serum (FBS), amino acids, vitamins and penicillin/streptomycin (Gibco; Scotland). Sodium pertechnetate (Na^99m^TcO_4_) solution was obtained from a commercial ^99^Mo/^99m^Tc generator (Radioisotope Division, Atomic Energy Organization of Iran).Analytical reverse phase high performance liquid chromatography (RP-HPLC) was performed on a HPLC system (Sykam S7131; Germany) equipped with a UV detector (Sykam S3210; Germany) set at L=280 nm, a flow-through gamma-detector (Raytest-Gabi; Germany) and aC18 analytical column (CC 250×4.6 Reprosil-pur ODS-3.5 µM). The mobile phase consisted of 0.1 % trifluoroacetic acid/water (Solvent A) and acetonitrile (Solvent B), and the gradient system used a flow rate of 1 mL/min in A:B ratio of: 95:5 at 0 min, 95:5 at 5 min,0:100 at 25 min, 0:100 at 27 min, 95:5 at 30 min, 95:5 at 35 min.A mass spectrometer (1100/BrukerDaltonic; Agilent; USA) with a VL instrument (LC/MS) was also used. Quantitative gamma countingwas performed on a well-type NaI gamma-counter (4001M MINIBIN; EG&GORTEC; USA).

*Synthesis*

The peptide was synthesized by standard Fmoc solid phase synthesis on Sieber amide resin with substitution, 0.65 mmol/g (25). Coupling of each amino acid was performed in the presence of 3 molar excess of Fmoc-amino acid, 3 molar excess of N-hydroxybenzotriazole (HOBt), 3 molar excess of diisopropylcarbodiimide (DIC) and 5 molar excess of diisopropylethylamine (DIPEA) in dimethylformamide (DMF). The Kaiser test determined the end of a coupling reaction and Fmoc groups were removed by adding 20% piperidine in DMF. The protecting groups of Mtt and 2-phenylisopropyl were removed by 1% TFA in advance of the peptide cyclization reaction, and protected peptide was cleaved from the resin by treating with a mixture of 2.5% of trifluoroacetic acid (TFA) and 5% of triisopropylsilane. Each protected peptide was cyclized by coupling the carboxylic group of the aspartic acid with the epsilon amino group of the lysine. After removing the organic solvents in vacuum, the crude product was precipitated with cold petroleum ether and diisopropyl ether (50:50 v/v).Then, the precipitate was dissolved in water/methanol (50:50) andpurified by semi-preparative RP-HPLC. The purified product was characterized by analytical HPLC and LC-MS.

^99m^ *Tc-Labeling of HYNIC-peptide via tricine*

A stock solution of HYNIC-peptide (1 mg/mL) was prepared by dissolving the peptide in distilled water. In order to optimize labeling efficiency, a series of studies as the following were performed: (i)varying the amount of tricine as a co-ligand in HYNIC labeling method, (ii) titrating the amount of SnCl_2_ as a reducing agent, (iii) changing the amount of HYNIC-peptide ligand, (iv) adjustment of the reaction pH and (v) radiolabeling conditions were optimized by varying temperature. Each parameter was examined carefully for its effects on radiolabeling yield of the final labeled compound. Radiolabeling of peptide was performed by adding 2–60 μL of the stock solution (2–60 μg of peptide) and 5–60 mg of tricine to 0.5 mL of water in a vial. Then 5–50 μg SnCl_2_ (5–50 μL of 1 mg/mL SnCl_2_, 2H_2_O in nitrogen-purged 0.1M HCl) was added to this solution. Finally, 1295 MBq of ^99m^TcO_4_^−^ in 0.5 mL saline was added to the solution and the pH [1-12] was adjusted. The vial was incubated in ambient temperature or at 90 °C for 10 min and then cooling down to ambient temperature. Labeling yield was checked on RP-HPLC using a flow-through gamma-detector by measuring the integration of peak areas.

*Stability in serum*

The stability of radiolabeled peptide in aqueous solution was evaluated by incubation of the reaction mixture at ambient temperature (25 °C) up to 24 h. Stability in human serum at 37 °C was tested in parallel after adding 100 μL of reaction mixture to 1mL of fresh human serum. The incubation mixtures were sampled at 1,6 and 24 h time points. Serum samples (100–200 μL) were treated with ethanol (200–400 μL) and centrifuged (4000 g, 5 min, 4 °C) to precipitate the serum proteins.20–100 μL aliquots from the supernatant were separated to assess the degradation of ^99m^Tc labeled peptide by RP-HPLC. 100 μL aliquots from the 25 °C incubated mixture were passed from a 0.22 μ filter to eliminate the probable ^99m^Tc colloids and then, 5–20 μL of filtrate were analyzed by RP-HPLC as well.

*Log p-values*

For determination of the partition coefficient, ^99m^Tc-HYNIC-peptide in PBS (0.5 mL) was mixed with 1-octanol (0.5 mL) in a 2-mL micro-tube. The tube was vigorously vortexed over a period of 5 min and centrifuged at 4,000×g for 5 minutes. Three aliquots were sampled (100 µL) from each layer andtheir radioactivity counted in a gamma counter. The average radioactivity values from each of the aqueous and octanol layers were used to calculate the octanol-to-water partition coefficient (P_o/w_) by dividing the radiolabeled peptide value (octanol phase) by the aqueous phase value.

*Cell culture*

The B16/F10 cells were cultured in RPMI-1640 supplemented with 10 % FBS, L-glutamine (2 mM), penicillin (100 units/mL) and streptomycin (100 µg/mL). Cells were maintained in a humidified 5 % CO_2_/air atmosphere at 37 °C. For all cell experiments, the cells were seeded in six well plates (1 million cells per well) and then incubated over-night with an internalization medium (RPMI-1640 with 1%FBS).

*Internalization*

Medium was removed from the six-well plates contain adhered B16/F10 cells, then the cells were washed once with medium (RPMI-1640 with 1% FBS; 2 mL). Furthermore, the same medium was added to each well, the plates were incubated at 37 °C for 1 h, and then ^99m^Tc-HYNIC-peptide (150 KBq; 2.5 pmol total peptide per well) was added.The cells were incubated at 37 °C for various time periods. To determine non-specific membrane binding, we also incubated cells with the radioligand in the presence of peptide (150 µL; 1 µmol/L).In separate experiments, the binding reaction was stopped at 0.5, 1, 2 and 4 h, respectively by removing medium from the adhered cells, washing them twice with cold phosphate-buffered saline (PBS; ~4 ^o^C; 1 mL), and then an acid wash for 10 min with cold glycine buffer (pH = 2.8; ~4 ^o^C; 1 mL) was performed twice. The last step was designed to take to distinguish between membrane-bound (acid releasable) and internalized (acid resistant) radioligand. Finally, the cells were treated with NaOH (1M). The activity of the culture medium and the receptor-bound and internalized fractions, both with and without cold peptide, were counted in a gamma-counter.

*Externalization*

Regarding to the release of the activity after maximal internalization and determination of the radioactivity which has remained in tumor cells the externalization of the maximal internalized activity was also measured. The B16/F10 cells (10^6^/well) were incubated with radioligand. After 2 h internalization at 37 °C and 5% CO_2_, the medium was removed and the cells were washed twice with 1 ml ice cold PBS. Acid wash for a period of 5 min twice with a glycine buffer of pH 2.8 was done to remove the receptor bound ligand. Cells were then incubated again at 37 °C with fresh internalization medium. After different time points (15, 30 min, 1, 2 and 4 h), the external mediums were removed for quantification of radioactivity in a gamma counter. The cells were solubilized in 1 N NaOH and removed, and the internalized radioactivity was quantified in a gamma counter. The externalized fraction was expressed as percentage of the total internalized amount per 1 million cells.

*Biodistribution*

Animal experiments were performed in compliance with the regulations of our institution and with generally accepted guidelines governing such work.Groups of three nude mice were used in each experiment. A suspension of B16/F10 cells (1×10^7^) in PBS was subcutaneously injected in the right flank of each nude mice. Seven days after inoculation, the tumors were inducted and then, ^99m^Tc-tricine-HYNIC-peptide (20 MBq) was injected via a femoral vein. Also a group of three animals were injected with peptide (100 g) in saline (0.9%; 50 L) as a co-injection with the radiopeptide (blocked animals). After 1, 4 and 24 h, the groups of mice were killed, then the organs of interest were excised, weighed and counted in the gamma counter. The percentage of the injected dose per gram (% ID/g) was calculated for each tissue. At 4 h after injection, accumulation of the tracer in tumor area was also assessed by planar scintigraphy under ether anesthesia.

## Results and discussion

MC1 receptors are known to be over-expressed in human skin melanomas (5,6). Based on this fact we assumed that targeting of the MC1 receptor with an optimized analogue of α-MSH would be very interesting for imaging of melanoma. The aim of this study wastarget the MC1 receptor *in-vitro *on a tumor cell line and *in-vivo* by a mouse tumor model, using an analogue of α-MSH containing a HYNIC-coupled lactam bridge-cyclic structure. Also, the optimum radiolabeling conditions was thoroughly investigated.

**Figure 1:**
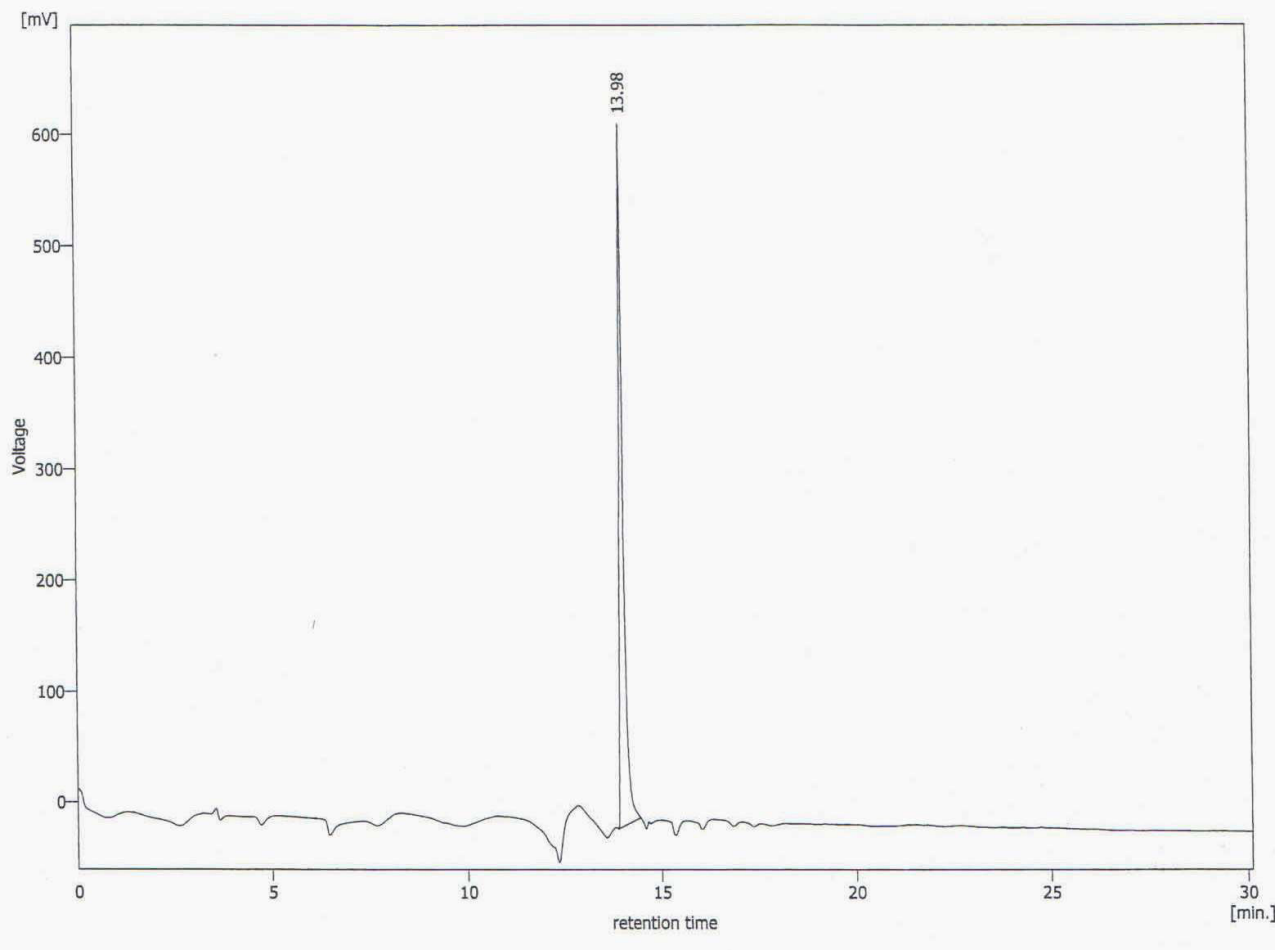
HPLC chromatogram of synthesized HYNIC-peptide with UV detector and L= 280nm (Rt = 13.98 and purity >98%).

**Figure 2:**
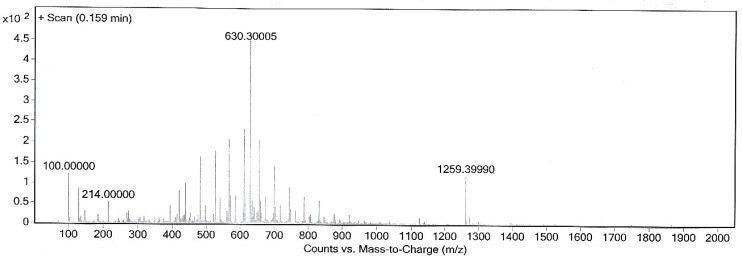
Mass spectrum of synthesized HYNIC-peptide.

This new [HYNIC-GABA-Nle]-CycMSH_hept_ derivative was conveniently synthesized by solid phase peptide synthesis on sieber amide resin via Fmoc strategy and it was obtained inan overall yield of 35% based on the removal of the first Fmoc group after cleavage, purification and lyophilization. The composition and structural identity of the purified HYNIC-peptide conjugate were verified by analytical HPLC and LC-MS (Table 1). The purity was >98% as confirmed by HPLC (Figure 1.). ESI-Mass analysis was consistent with the calculated molecular weight for the HYNIC-peptide (Figure 2.). 

A variety of bifunctional chelating agents (BFCA) have been used to label proteins, peptides, and other biologically active molecules by ^99m^Tc (26-28).More recently HYNIC became a more popular BFCA because high specific activity products could be prepared, in the presence of various co-ligands that had an effect on the hydrophilicity and pharmacokinetics of radiopeptide (22,23,29 and 30). Of the different co-ligands, tricine achieves the best radiolabeling efficiency.

**Figure 3:**
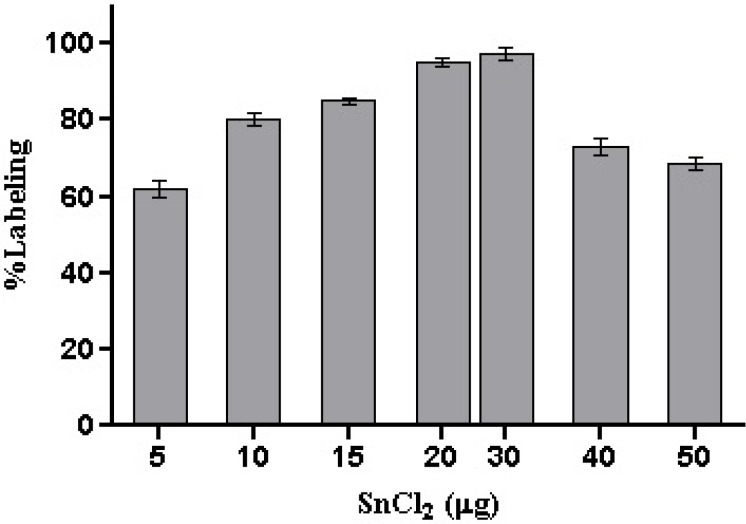
Effect of SnCl_2_ on labeling yield of ^99m^Tc-HYNIC-peptide (mean ± SD, n=3).

To develop a ^99m^Tc-HYNIC-peptide, an optimal combination of individual ingredients including peptide ligand, SnCl_2_ and tricine were systematically examined. As shown in Figure 3. and Table. 2. The amount of SnCl_2_ and tricine affect the labeling yield of ^99m^Tc-HYNIC-peptide, respectively. The optimal amounts required of SnCl_2_ and tricine were found to be 20-30 μg of SnCl_2_ and 10-20 mg of tricine. A stable radioligand with radiochemical purity of more than 98% could be obtained by using a combination of 30 μg SnCl_2_ and 20 mg of tricine.The amount of peptide used in the labeling also is important, because the excess of free ligand may lead to an increased risk ofinducing pharmacologically undesired side effects and alsodecreasing the specific activity of labeled ligand. Therefore, reducing the amount of ligand used in the formulation is desirable. As was shown in Table 3. by lowering the amount of peptide to 8 μg a labeling yield of greater than 96% can besuccessfully reached. A significant reduction of labeling yieldby further reducing the amount of free ligand was observed. No significant improvement of radiochemical purityobserved while concentration of peptide exceeded 8 μg. On the other hand, by increasing the amount of peptide from 2 to 60 μg, specific activities decreased from 815.0 to 27.2 MBq, respectively. On the basis of these observations, maximum amount of HYNIC-peptide up to 10 μg was used for the labeling to insurepresence of sufficient amount of ligand for complex formation.

**Figure 4:**
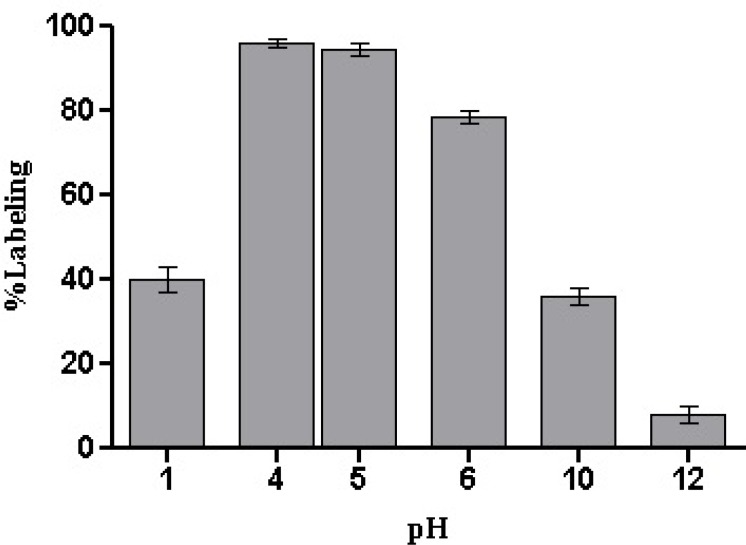
Effect of reaction pH on ^99m^Tc-HYNIC-peptide labeling yield (mean ± SD, n=3).

The effect of reaction pH was also investigated and theoptimal pH range to produce a high labeling yield of^99m^Tc-HYNIC-peptide was found to be around 4–5 (without any additional pH adjustment) (Figure 4.). Either end of pH ranges willreduce the labeling yield (40% at pH = 1 and 8% at pH=12).

Also when labeling reaction was carried out by heating the mixture at 90 °C for 10 min, labeling yield of >98% was observedin contrast to ambient temperature condition in which the labelingyield was <90%.

**Figure 5:**
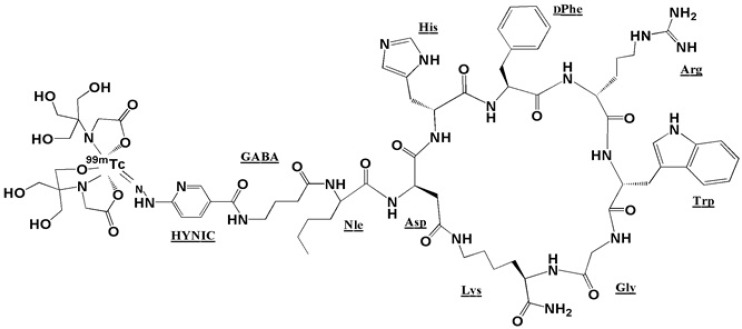
A proposed structure of [HYNIC-GABA-Nle-CycMSH_hept_] after ^99m^Tc labeling in a tricine co-ligand system.

**Figure 6:**
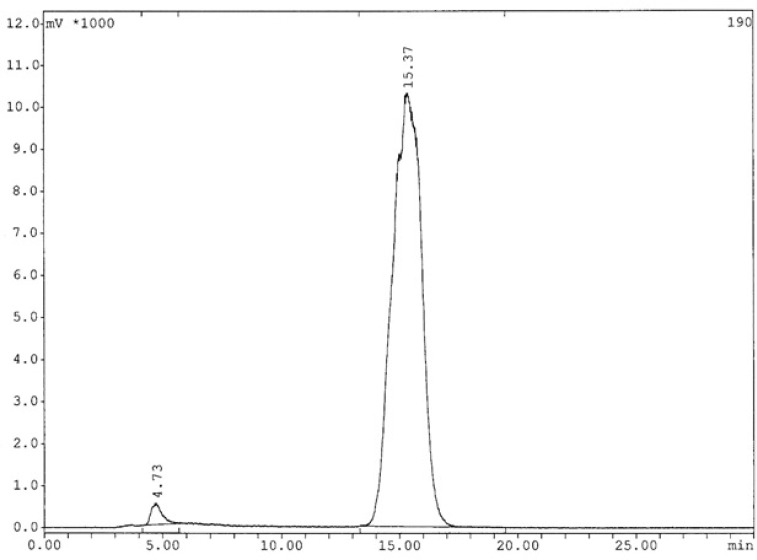
Radiochromatogram of ^99m^Tc-labeled HYNIC-peptide using tricine as a co-ligand after 6h (The retention time for radiopeptide: 15.37 min).

**Figure 7:**
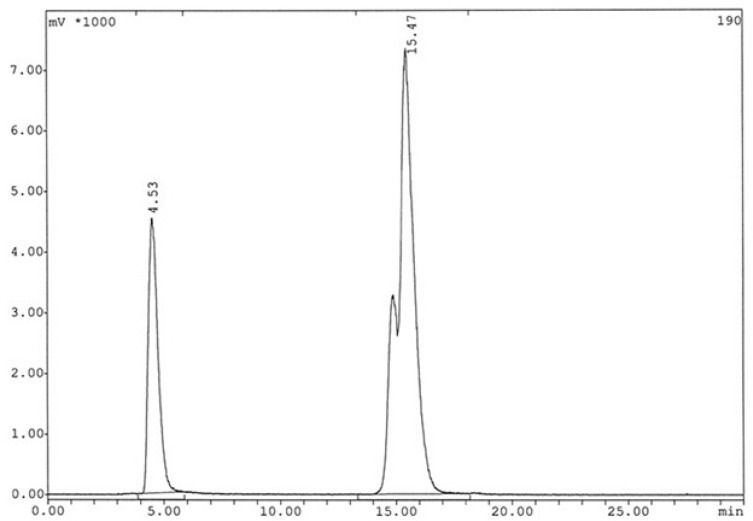
Radiochromatogram of ^99m^Tc-labeled HYNIC-peptide using tricine as a co-ligand after 24 h (The retention time for radiopeptide: 15.47 min).

**Figure 8:**
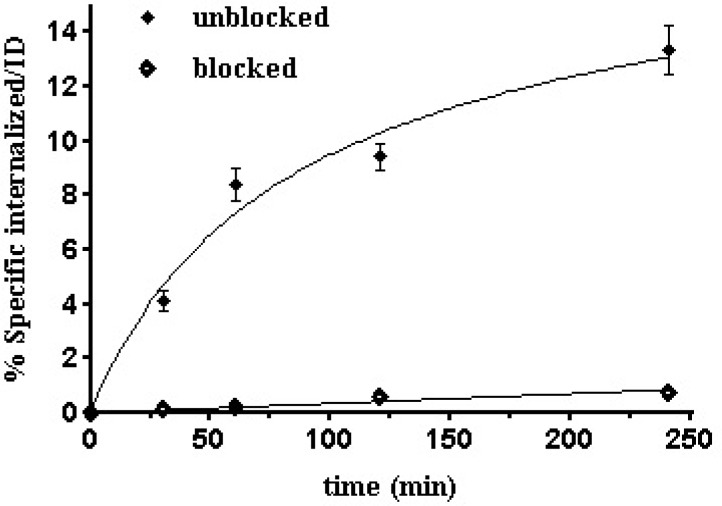
Internalization rate of ^99m^Tc-Tricin-HYNIC-peptideinto B16/F10 cells. Data are from three independent experiments with triplicates in each experiment and are expressed as specific internalization

**Figure 9:**
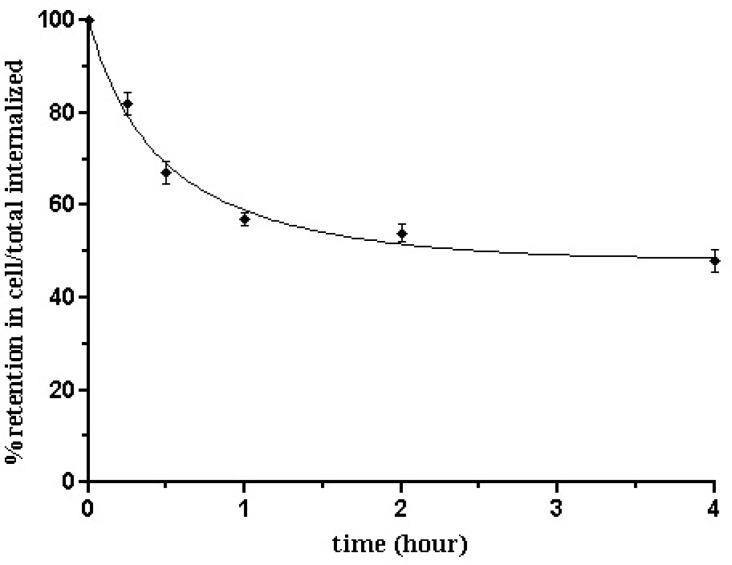
Externalization over time for ^99m^Tc-Tricin-HYNIC-peptide into B16/F10 cells. Data result from two independent experiments with triplicates in each experimentand are expressed as percentage of total internalized amount

**Figure 10:**
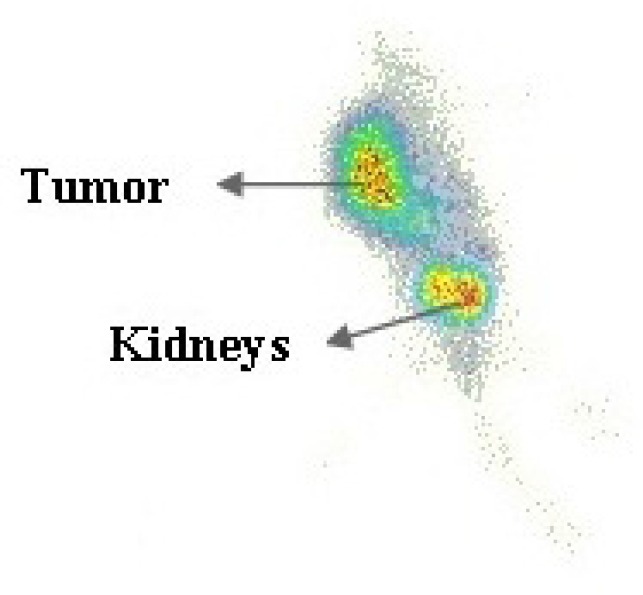
Scintigraphy image of tumor bearing nude mice 4h after injection of ^99m^Tc-HYNIC-peptide, which shows tumor and kidneys.

**Table 1:** Demographic data and baseline medical and medication history of the study groups

Variable	Subgroup	Intervention group	Control group	p value
Age (year)	N/A	50.38 ± 12.25+	51.48 ± 12.49	0.74
Sex	Male	5 (17.24%)	3 (11.11%)	0.71
	Female	24 (82.76%)	24 (88.88%)	
RA history (years)	N/A	6.69 ± 4.44 [6.00, 4.00-8.50]*	7.09 ± 5.20 [6.00, 3.00-12.00]	0.98
Main referral reason	New case	3 (10.34%)	4 (14.81%)	0.81
	Flare-up	13 (44.83%)	10 (37.04%)	
	Follow up	13 (44.83%)	13 (48.15%)	
Concurrent rheumatoid disease	Yes	9 (31.03%)	9 (33.33%)	0.85
	No	20 (68.97%)	18 (66.67%)	
Comorbidity	Yes	8 (27.59%)	10 (37.04%)	0.45
	No	21 (72.41%)	17 (62.96%)	
Concurrent drug	Yes	8 (27.59%)	7 (25.93%)	0.89
	No	21 (72.41%)	20 (74.07)	
No of tender joints	N/A	[3.00, 2.00-6.50]	[3.00, 1.00-4.00]	0.24
No of swollen joints	N/A	[3.00, 2.00-5.00]	[2.00, 1.00-4.00]	0.07
Patient global assessment	N/A	81.38 ± 19.95 [80.00, 70.00-100.00]	79.26 ± 23.85 [80.00, 50.00-100.00]	0.98
ESR	N/A	26.41 ± 19.78 [21.00, 12.00-33.50]	21.63 ± 17.38 [15.00, 10.00-32.00]	0.23
DAS28_ESR	N/A	4.81 ± 1.00	4.31 ± 1.05	0.07
Menopause female patients	Yes	16 (66.67%)	15 (62.50%)	0.76
	No	8 (33.33%)	9 (37.50%)	

N/A: Not Applicable; RA: Rheumatoid Arthritis; **+** Continuous data are presented as mean ± standard deviation;

* [Median, Interquartile range] for discrete or non-normally distributed variables; ESR: Erythrocyte Sedimentation Rate.

**Table 2:** Results of the repeated measures ANOVA

**Remission indicator**	**Repeated Measures ANOVA Result**	
	**Multivariate** **p value**	**Within-subjects contrasts** **p value**
DAS28_ESR	0.55	0.33
No of Tender joints	0.60	0.84
No of swollen joints	0.68	0.81
ESR	0.43	0.29
PtGA	0.25	0.03

ANOVA: Analysis of Variance; DAS: Disease Activity Score with 28-joint counts; ESR: Erythrocyte Sedimentation Rate; PtGA: Patient Global Assessment of disease activity

**Table 3:** Results of the comparison between reduction (baseline - week 16) in the measures of RA remission in the control and intervention groups.

**Remission indicator**	**Intervention group**	**Control group**	**p value**
DAS28_ESR	1.76 ± 1.39^+^	1.37 ± 1.35	0.34
No of Tender joints	[2.00, 1.00-4.00]*	[2.00, 0.00-3.00]	0.49
No of swollen joints	[2.00, 1.00-3.25]	[1, 0.00-3.00]	0.45
ESR	7.65 ± 16.16	3.57 ± 11.90	0.33
PtGA	44.09 ± 30.03	25.22 ± 26.26	0.03

DAS: Disease Activity Score with 28-joint counts; ESR: Erythrocyte Sedimentation Rate; PtGA: Patient Global Assessment of disease activity; + Continuous data are presented as mean ± standard deviation;

* [Median, Interquartile range] for discrete or non-normally distributed variables.

Combining the previous results for acquiring maximum complexation yield with highest possible specific activity showed that by using 10 μg HYNIC-peptide as a ligand, 20 mg tricine as a co-ligand, and 30 μg stannous chloride dihydrate as reducing agent at pH = 4.5, a high yield (>98%) and a specific activity of 163MBq/nmol for radioligand could be reached. The proposed structure for desired complex is shown in Figure 5. 

The analysis results by RP-HPLC (Figure 6.) show labeling yield of >98%at 15.37min retention time for ^99m^Tc-tricine-HYNIC-peptide. Also, stability studies in aqueous solution and human serum showed radiolabeled complex with no significant release of ^99m^TcO_4_^−^ or peptide degradation up to 6 h, but in 24 h, labeling yield reduce to 75% (Figure 7.). It has been reported that with tricine as a co-ligand,^99m^Tc-tricine-complex was not stable, particularly in dilute solutions, because of the different bonding modalities of the hydrazine moiety of the HYNIC and the tricineco-ligand (31, 32).

Protein binding of radiolabeled peptide was measured by precipitationmethod resulting 37%forradioconjugate. Plasma proteins binding could be due to transchelation, which is possible with exchange reactions among co-ligands and the possible action of HYNIC as a mono- or bi-dentated ligand complexing ^99m^Tc. Previous studies have also suggested that similar affects such as the exchange reactions of tricine co-ligand with proteins in plasma and lysosomes may occur (33, 34).

The calculated partition coefficient for labeled compound was (log P = −1.31 ± 0.12 %), which is a good indicator of its high hydrophilicity.

Figure 8. shows the results of the test for specific internalization of the radioligand into B16/F10 cells as a function of time. The specific cell uptake after 1 h was 8.40 ± 0.6%,which increased to 13.35±0.9% after 4 h.This specific internalization was not unexpected because α-MSHsequence offers agonistic property to the compound.Previous studies in a series of ^99m^Tc-labeled cyclic-MSH derivatives have demonstrated internalization and receptor mediated trapping of labeled compounds (17,35 and 36). This finding could be a result of the receptor mediated endocytosis mechanism which also called clathrin-dependent endocytosis (CME). As known, binding of peptide to MC1 receptor leads to internalization by CME mechanism to the acidified endosomes where the complex dissociates. Consequently, peptide is degraded and MC1 receptor is re-expressed on the cell surface (37, 38). CME could be the responsible mechanism for internalization of ^99m^Tc labeled HYNIC-peptide on B16/F10 cells in the same way. The internalization ability of a radioligand is important to make it the ideal as it can no longer take part in the equilibrium process and also it guarantees intracellular delivery of the radioisotope (39). Differences in uptake between blocked and unblocked cells at various time periods are very noticeable (*p<0.05).Considering its fast and receptorspecific internalization of this radiopeptide as it was demonstratedin its uptake results in MC1 receptor blocked cells experiments, is anindication that binding properties of this radiopeptide is not affected by modification and labeling procedures.

Besides efflux curve (Figure 9.) of ^99m^Tc-tricine- HYNIC-peptide in B16/F10 cells after 2 h of internalization showed an acceptable intracellular trapping. After 15 min, 18.46 ± 1.13% of radioactivity was externalized, and this amount increased to 51.32 ± 2.14 % up to 4 h which was the maximum and after that the percentage of externalization reaches a plateau. In other words approximately 50% of the internalized activity remained inside the cells after 4 h. In comparison, a less stable compound shows a significant decrease of internalized activity at the very first hours. It can be due to a rapid degradation of the compound in the tumor cells. So, the more stable the compound, the higher the radioactivity retention.

Results from biodistribution studies using the ^99m^Tc-tricine- HYNIC-peptide are presented in Table 4. The data were expressed as the percentage of injected dose per gram of tissue (% ID/g) based on the previous reported studies (40). The highest uptake was observed in kidneys (7.38 ± 0.56% ID/g at 1 h after injection) and generally, a rapid elimination via the urinary tract could be observed. Uptake values in liver and heart were lower than 2.0% ID/g which confirms the hydrophilicity of the radiolabeled peptide. 

In overall, the rapid renal excretion of activity which was observed for this peptide conjugate resulted in the lowest values in blood (1.26 ± 0.13% ID/g at 1 h), liver (1.48 ± 0.08% ID/g at 1 h), and intestines (0.65±0.05% ID/g at 1 h). So, it should be noticed that the main percentage of the total dose injected is located very early in the kidneys. This is also in good agreement with the calculated partition coefficient of the labeled compound (log P = −1.31 ± 0.12%), showing high hydrophilic character for the radiopeptide.

Aside from the kidneys, accumulation of radiopeptide in MC1 receptor positive tissues like the tumor was observed. The uptake in tumor was specific and receptor mediated, as shown by the lower uptake after co-injection of cold peptide. Tumor uptake was significantly reduced from 5.20% ID/g in the control group (un-blocked) to 2.33 % ID/g in the blocked group. On the other hand, the uptake reduction in non-targeted tissues due to blocking was not significant. The tumor accumulation of this radioconjugate and its efficient pharmacokinetic behavior such as low tendency to accumulate in liver followed by its high kidney excretion due to low lipophilicity are the major advantages of this compound.

The tumor location could be visualized through scintigraphy within 4 h after injection which confirms the specific uptake of radiopeptide by the tumor (Figure 10.). The mainly renal excretion at the early stages is an attractive pharmacokinetic behavior for a diagnostic radiopharmaceutical of interest.

## Conclusion

The conjugate of ^99m^Tc-tricine-HYNIC-GABANle-CycMSH_hept _analog was prepared by labeling of peptide, HYNIC and tricine as a co-ligand demonstrated an excellent radiochemical stability even up to 6 h post labeling. The prepared conjugate showed high accumulation in tumor as a positive MC1 receptor targeted tissue followed by excretion via the kidney. These promising characteristics make the new designed labeled peptide conjugate a suitable candidate for diagnosis of metastaticmelanomas.
